# Interplay between fatty acid desaturase2 (*FADS*2) rs174583 genetic variant and dietary antioxidant capacity: cardio-metabolic risk factors in obese individuals

**DOI:** 10.1186/s12902-022-01075-7

**Published:** 2022-06-30

**Authors:** Mahdieh Khodarahmi, Parisa Javidzade, Mahdieh Abbasalizad Farhangi, Ahmad Hashemzehi, Houman Kahroba

**Affiliations:** 1 Department of Community Nutrition, Faculty of Nutrition and Food Science, Tabriz University of Medical Sciences, Tabriz, Iran; 2 Department of Genetics, Faculty of Science, Shahid Chamran University of Ahvaz, Ahvaz, Iran; 3 Drug Applied Research Center, Tabriz University of Medical Sciences, Attar-Neishabouri Ave, Golgasht St, Tabriz, 5165665931 Iran; 4 Department of Pharmaceutics, Faculty of Pharmacy, Tehran University of Medical Sciences, Tehran, Iran; 5 Department of Toxicogenomics, GROW School of Oncology and Development Biology, Maastricht University, Maastricht, the Netherlands; 6 Centre for Environmental Sciences, Hasselt University, Hasselt, Belgium

**Keywords:** Non-enzymatic antioxidant capacity (NEAC), Obesity, Gene-diet interaction, Fatty acid desaturase (*FADS*) gene, Cardio-metabolic risk

## Abstract

**Objective:**

Polymorphisms of the fatty acid desaturase (*FADS*) gene cluster have been associated with obesity and its-related consequences. This cross-sectional study aimed to investigate whether the adherence to dietary non-enzymatic antioxidant capacity (NEAC), reflecting the antioxidant potential of the whole diet, modifies the association of *FADS*2 rs174583 polymorphism with cardio-metabolic risk factors in obese adults.

**Methods:**

The present study included 347 healthy obese adults (aged 20–50 years). Dietary NEAC was assessed by a validated food frequency questionnaire with 147 items and estimated through total radical-trapping antioxidant parameters (TRAP), oxygen radical absorbance capacity (ORAC), and ferric reducing ability of plasma (FRAP) with the use of published databases. *FADS*2 rs174583 polymorphism was characterized using PCR–RFLP. ANCOVA multivariate interaction model was used to analyze gene-diet interactions.

**Results:**

after adjustment for the confounding variables (age, physical activity, SES and WC), this study showed significant interactions between rs174583 polymorphism and adherence to dietary ORAC on the serum cholesterol (*P*_Interaction_ = 0.029), LDL-C (*P*_Interaction_ = 0.025) and HDL-C levels (*P*_Interaction_ = 0.049) among the male group; minor allele carriers who had the highest adherence to the NEAC (ORAC) showed a better metabolic profile (lower TG and LDL-C and higher HDL-C) (*P* < 0.05). Among women, the dietary ORAC-rs174583 interactions were statistically significant for the serum insulin concentration (*P*_Interaction_ = 0.020), QUICKI (*P*_Interaction_ = 0.023) and HOMA-IR (*P*_Interaction_ = 0.017); the highest QUICKI and the lowest HOMA-IR and serum insulin levels were observed in the CC homozygote carriers with the moderate compliance with the dietary ORAC (*P* < 0.05). In addition, the dietary TRAP modified the association between *FADS*2 variant and change in LDL-C levels (*P*_Interaction_ = 0.037); the homozygous wild-type (CC) women who placed in the top tertile of TRAP had significantly the lowest LDL-C levels than those in the second tertile (*P* < 0.05).

**Conclusion:**

These data indicate that the *FADS*2 rs174583 polymorphism interacts with the dietary NEAC to influence cardio-metabolic risk factors in obese subjects. Replication in prospective cohort studies among other populations is required to confirm the results of our study.

**Supplementary Information:**

The online version contains supplementary material available at 10.1186/s12902-022-01075-7.

## What is already known about this topic?

The role of fatty acid desaturase 2 genetic variants in promoting obesity and obesity-related disorders like insulin resistance and dyslipidemia is reported in previous studies. However, it is unknown that how dietary indices particularly dietary antioxidants can alleviate the role of these genetic variants in the obesity-related comorbidities.

## What does this article add?

In the current work, for the first time, we evaluated the effects of dietary non-enzymatic antioxidant capacity (NEAC) on cardio-metabolic risk factors among different genotypes of the fatty acid desaturase 2 genetic variant in obese individuals to further highlight the role of dietary antioxidant indices in prevention of genetic susceptibility to obesity-associated disorders.

## Introduction

Obesity, as a major global health problem, is increasing at an alarming rate worldwide [1]. It has been estimated that globally, more than 13% of the world's adult population are obese. Similarly, based on available national data, approximately 22% of Iranian adults were affected by obesity in 2016 [2]. There is accumulating evidence showing that plasma fatty acids composition has significant effects on development of obesity-related non-communicable diseases [3]. On the other hand, both experimental and clinical studies have suggested that oxidative stress which is characterized by reduced antioxidant capacity and/or overproduction of reactive oxygen species (ROS) plays an important role in the development of obesity-related health outcomes [4].

Obesity is a multifactorial abnormality caused by both environmental and genetic factors and complex interactions between them [5]. Diet, as a key environmental factor, can interact with genetic background to affect the susceptibility to plenty of diseases [6]. Several studies have provided evidence that intake of dietary compounds with antioxidant activity is inversely associated with oxidative stress–induced conditions such as obesity [7]. Recently, the concept of dietary total antioxidant capacity (TAC) has been introduced to estimate the cumulative effects of antioxidants in the overall diet [8]. Dietary non-enzymatic total antioxidant capacity (NEAC), also known as TAC, can be measured through different chemical assays: oxygen radical absorbance capacity (ORAC), ferric reducing ability of plasma (FRAP) and total radical-trapping antioxidant parameters (TRAP) [9]. Numerous studies have indicated that dietary NEAC values are inversely related to cardio-metabolic risk factors [10] and other diet-related non-communicable diseases such as cardiovascular disease (CVD) [11], type 2 diabetes (T2D) [12], and cancers [13]. Nevertheless, the current evidence with regard to the relationship between NEAC and health outcomes is far from conclusive [14]. In addition, research regarding the effects of dietary NEAC on health outcomes has mostly been carried out in the western countries [15] and limited information is available from the middle-eastern populations [16].

Interestingly, fatty acids status has been related to the risk of multiple diet-related chronic diseases [17] and, accordingly, the determinants of fatty acid metabolism such as genetic variants in fatty acid desaturases (*FADS*) should be completely understood. Recently, genome wide association studies (GWAS) have indicated that polymorphisms in the *FADS* gene cluster have a main effect on obesity and other metabolic diseases [18, 19]. The *FADS*1 and *FADS*2 genes, located closely on the chromosome 11 (11q12–13.1), encode delta-5-desaturase (D5D) and delta-6-desaturase (D6D), respectively; the essential enzymes involved in the homeostasis of polyunsaturated fatty acids (PUFA) [20, 21]. Reports indicate that changes in the activity of D5D and D6D enzymes can lead to alteration in the profile of endogenous fatty acids and subsequently, development of non-communicable diseases, such as obesity, T2D, metabolic syndrome (MetS), and CVDs [22, 23]. A number of recent studies have revealed that the activities of these enzymes can be affected by single‐nucleotide polymorphisms (SNPs) of *FADS*1 and *FADS*2 genes [24, 25]. However, the results of studies are not consistent enough to approve the outcomes of SNP association studies; this suggests that interactions between genetic and environmental factors such as diet may be influential. On the other hand, a number of studies have demonstrated that oxidative stress is involved in the pathogenesis of psychiatric diseases which may cause development of obesity and its-related metabolic complications [26]. Therefore, a diet high in antioxidants may protect against oxidative stress and result in improvement of the mental health [27]. In other words, obesity-related consequences are affected by the interactions between psychological parameters, obesogenic environment such as unhealthy dietary intakes and sedentary lifestyle [28]. Hence, assessment of gene-diet interactions is important as it helps to generate individualized effective dietary strategies [29]. So, we aimed to examine how dietary antioxidant capacity interacts with genetic variant of *FADS*2 (rs174583) in relation to changes in cardio-metabolic risk factors of obese adults.

## Materials and methods

### Participants

This cross-sectional study was conducted in Tabriz city, one of the major cities in the northwest of Iran, during November 2017 to October 2018. Individuals were enrolled using convenience sampling method through announcements and posters placed in public areas of the city. Participants were included if they met the following criteria: good health, obesity (body mass index (BMI) ≥ 30) and ages of 20–50 years. At the beginning of the study, 400 participants were willing to participate in the study. The exclusion criteria were pregnancy, lactation, menopause, a history of CVDs, T2D, cancer, renal disease, hypertension, hyperlipidemia and hepatic disorders or taking any medications and supplements which influence weight and variables studied such as loop diuretics, cortico-steroids, antidepressants, antihypertensive agents and statins, any recent surgery such as bariatric, and daily energy intake outside the range of 800–4200 kcal/day [30, 31]. Finally, all these exclusions left 347 subjects for analysis. To calculate the sample size, the association between dietary quality indices and obesity was considered as a key dependent variable. For this purpose, with regard to the correlation coefficient (r) of 0.25 [32], α = 0.05 and power of 80%, using G-power software, the minimum sample size was estimated at 160. To perform sex-stratified analyses, the final sample size of 340 was considered for our study. Written informed consent was obtained from each participant before taking part in this study and the study protocol was approved by the ethics committee of the Tabriz University of Medical Sciences (registration code IR.TBZMED.REC.1400.889).

### Definition of MetS

The presence of MetS was identified based on criteria established by the Iranian National Committee of Obesity [33]. Participants with three or more of the following criteria were considered to have MetS: waist circumference > 95 cm (men and women), high blood pressure (systolic blood pressure (SBP) ⩾130 mmHg or diastolic blood pressure (DBP) ⩾85 mmHg, fasting triglyceride (TG) level ⩾150 mg/dl, fasting high-density lipoprotein cholesterol (HDL-C) level less than 40 mg/dl (men) or 50 mg/dl (women), and fasting blood sugar ⩾100 mg/dl.

### Dietary intake and dietary non-enzymatic antioxidant capacity assessment

Usual dietary intake during the previous year was assessed via face-to-face interviews using a valid and reliable 147-items semi-quantitative food-frequency questionnaire (FFQ) [34, 35]. All information was collected by trained dietitians. Participants were asked to report their frequency and amount of the intake of each food item during the last year based on a daily, weekly, monthly basis and then by using household measures, portion sizes of consumed foods were converted to gram/day. Daily intake of energy and nutrients collected through the FFQ were analyzed using Iranian Food Composition Table (FCT) [35] and complemented with the USDA FCT [36].

The values of NEAC, as a marker of dietary antioxidant potential, were calculated using the following indicators [9, 37, 38]: FRAP which measures the in vitro reduction of the ferric ion to ferrous ion in the presence of antioxidants, TRAP which measures the chain-breaking antioxidant potential to scavenge peroxyl radicals and ORAC that estimates the antioxidant capacity against peroxyl radicals by measuring the area under the curve of radical-induced fluorescence decay. Since there was no available database to calculate the quantity of antioxidants in Iranian foods, ORAC, FRAP and TRAP values assigned to each food item were obtained from previously published databases [37, 39, 40]. We assigned the NEAC for 100, 59, and 57 food items in the FFQ by ORAC, FRAP, and TRAP, respectively. We calculated dietary NEAC without the contribution of coffee since it remains unclear whether the main contributors to the in vitro antioxidant capacity of coffee; the Maillard products from the coffee roasting process, are absorbed efficiently due to their high molecular weight and if the same antioxidant activity is displayed in vivo [41]. To calculate total daily dietary NEAC for every participant, the frequency of consumption of each food item was multiplied by its corresponding NEAC values and, then, they were summed up. Subjects were categorized based on tertiles of ORAC, FRAP and TRAP.

### Socio-demographic, blood pressure and anthropometric measurements

General information such as age, gender, marital status, smoking, medical history, and socioeconomic status (SES) was collected using questionnaires which were administered to individuals by face-to-face interviews [42]. SES was determined through the following questions: educational status, occupational position, house ownership, and family size which were considered as individual indicators. In the current study, education was measured as a categorical variable where participants were asked to specify their highest level of educational attainment. This variable was recorded on a 5-point scale ranging from 0 to 5 (illiterate: 0, less than diploma: 1, diploma and associate degree: 2, bachelors: 3, masters: 4 and higher: 5). Female subjects’ occupational class was categorized into five groups (housewife, employee, student, self-employed and others). Occupational status of male subjects was categorized as follow: unemployed: 1, worker, farmer and rancher: 2, others: 3, employee: 4 and self-employed: 5. Accordingly, participants were categorized as ≤ 3, 4–5, ≥ 6 in terms of family size. Besides, they were given scores 1 and 2 if they were tenant and landlord, respectively. Next, each participant received a score between zero and 15 for the whole SES score and, then, individuals were classified into 3 categories: low, middle, and high according to SES tertiles. A short version of the International Physical Activity Questionnaire (IPAQ) was used to assess the physical activity level of participants [43]. Body weight of participants was measured in light clothing using a Seca scale (Seca, Germany) with an accuracy of 0.1 kg. A tape measure with a precision of 0.1 cm was used to measure height while subjects were standing in the normal position without shoes. Participants’ body composition measurements were conducted through bioelectrical impedance analysis (BIA) technology (Tanita, BC-418 MA, Tokyo, Japan). Waist circumference was obtained in the slimmest area while participants were at the end of a normal exhalation, using an unscratched tape and was recorded to the nearest 0.1 cm. SBP and DBP were measured using a mercury sphygmomanometer twice, after 15 min rest in a sitting position and finally, the average of the two measurements was recorded.

### Mental health and appetite assessments

The severity of the various mental disorders was determined using a validated self-administered the Depression, Anxiety and Stress Scale-21 Items (DASS-21) questionnaire [44, 45]. The Cronbach’s alpha (internal consistency) for the DASS questionnaire in Iranian population has been reported as follows: 0.77 for depression, 0.79 for anxiety, and 0.78 for stress [44]. This questionnaire consists of 21 items comprising 3 subscales of 7 items which assess the emotional states of depression, anxiety and stress over the last week. Each item in this instrument was rated based on a Likert scale from 0 “did not apply to me at all” to 3 “applied to me very much or most of the time” through individual structured interviews with the subjects. The related items scores for each subscale were summed and multiplied by a factor 2 to give a total score that ranges from 0 to 42 and then participants were divided into 5 categories: normal, mild, moderate, severe and extremely severe, according to cut-off scores which have been proposed by Lovibond and Lovibond [46]. Higher subscale scores reflect more severe psychological disorders.

To assess participant’s appetite level, a 10-cm visual analog scale (VAS) questionnaire, with prior evidence of validity and reliability, was applied [47]. This tool includes different questions about feeling of hunger, satiation, fullness, prospective food consumption, thirst, and the desire to eat something sweet, salty, or fat. Participants were asked to complete this questionnaire by making a mark across a 100 mm horizontal line corresponding to their feelings and, subsequently, VAS score was determined by measuring the distance from the left side of the line to the mark. For the rest of information about technical methodology, see the supplementary data.

### Statistical analysis

Normal distribution of data was checked by descriptive measures such as coefficients of skewness and kurtosis, mean and standard deviation [48]. Data were presented as means ± standard deviations for normally distributed quantitative variables, the median (25th and 75th percentile) for variables with skewed distributions and the frequency (%) for discrete variables. The comparison of categorical variables was performed by Chi-square test. Quantitative variables with normal and non-normal distribution were compared with Analysis of variance (ANOVA) and the Kruskal–Wallis tests, respectively. Sex-stratified multivariable multinomial logistic regression analysis was applied to test the associations between dietary NEAC and rs174583 polymorphism of *FADS*2 gene. The potential interactions between *FADS*2 polymorphism (rs174583) and dietary NEAC on cardio-metabolic risk factors based on sex groups were examined by ANCOVA multivariate interaction model, after controlling for confounding variables (age, physical activity, SES and WC). Since the interaction effects are difficult to explain, all significant interactions were depicted as graphs to help their interpretations. All statistical analyses were conducted using the Statistical Package for Social Sciences (SPSS, Inc., Chicago, IL, version 21). A *P*-value less than 0.05 was considered to be statistically significant in all analyses.

## Results

### Study population characteristics

Briefly, after application of the exclusion criteria, 53 potential subjects were excluded from the study and, consequently, 347 healthy obese adults (58.2% male, 41.8% female) aged 20 to 50 years were recruited.

### Comparisons between *FADS*2 rs174583 genotypes

The general characteristics of participants based on *FADS* rs174583 genotypes are provided in Table 1. No significant differences were found regarding anthropometric, socio-demographic, dietary and mental health parameters across *FADS*2 rs174583 genotypes; neither in men nor in women.

**Table 1:** Characteristics of participants according to the *FADS*2 rs174583 genotypes

	**Men**				**Women**			
	**CC**	**CT**	**TT**	***P***^a^	**CC**	**CT**	**TT**	***P***^a^
**Age (y)**	39.06 (7.55)	37.03 (6.34)	41.20 (5.20)	0.168	37.42 (7.20)	37.43 (7.91)	36.50 (8.14)	0.961
**Weight (kg)**	103.16 (10.26)	101.43 (10.63)	101.59 (14.18)	0.783	88.79 (13.58)	88.64 (12.28)	94.82 (7.50)	0.517
**WC (cm)**	113.25 (7.11)	112.46 (7.82)	114.00 (10.17)	0.827	102.75 (9.27)	101.38 (9.51)	105.67 (9.18)	0.083
**FM (kg)**	29.61 (7.19)	28.63 (6.95)	31.16 (8.97)	0.597	38.50 (8.76)	37.90 (8.80)	41.73 (4.34)	0.590
**FFM (kg)**	73.61 (5.65)	72.82 (6.17)	70.43 (6.51)	0.340	50.30 (5.84)	50.76 (4.53)	53.08 (4.08)	0.473
**Appetite**	35.29 (10.21)	35.41 (10.24)	31.40 (7.73)	0.503	32.58 (8.95)	31.78 (8.06)	33.50 (8.34)	0.862
**Physical activity level, (%)**				0.921				0.225
Low	40.0	33.3	30.0		62.5	57.1	100.0	
Moderate	25.7	38.5	40.0		16.7	31.0	0.0	
High	34.3	28.2	30.0		20.8	11.9	0.0	
**Marital status, (%)**				0.844				0.569
Married	82.9	82.1	80.0		79.2	90.5	83.3	
Single	17.1	17.9	20.0		20.8	9.5	16.7	
**SES, (%)**				0.973				0.827
Low	0.0	0.0	0.0		4.2	7.1	0.0	
Middle	38.2	25.6	50.0		83.3	76.2	83.3	
High	61.8	74.4	50.0		12.5	16.7	16.7	
**Stress, (%)**				0.663				0.501
Normal	48.6	38.5	40.0		29.2	33.3	50.0	
Mild	5.7	7.7	10.0		20.8	14.3	16.7	
Moderate	17.1	23.1	20.0		33.3	28.6	33.3	
Severe	17.1	15.4	20.0		12.5	23.8	0.0	
Extremely severe	11.4	15.4	10.0		4.2	0.0	0.0	
**Anxiety, (%)**				0.666				0.250
Normal	48.6	38.5	40.0		45.8	21.4	16.7	
Mild	5.8	7.6	10.0		12.5	11.9	16.7	
Moderate	17.1	23.1	20.0		16.7	31.0	50.0	
Severe	17.1	15.4	20.0		8.3	14.3	16.7	
Extremely-severe	11.4	15.4	10.0		16.7	21.4	0.0	
**Depression, (%)**				0.104				0.431
Normal	45.7	53.8	80.0		29.2	35.7	50.0	
Mild	8.6	12.8	10.0		12.5	19.0	0.0	
Moderate	28.6	20.5	0.0		25.0	26.2	33.3	
Severe	8.6	7.7	0.0		25.0	7.1	0.0	
Extremely severe	8.6	5.1	10.0		8.3	11.9	16.7	
**ORAC (µmol TE/d)**	16,177.95 (12,781.03, 23,482.19)	17,133.49 (12,591.73, 23,604.74)	19,642.65 (9,936.24, 37,167.90)	0.948	24,658.08 (14,030.06, 29,422.07)	20,017.15 (16,051.33, 29,259.28)	19,565.08 (15,060.64, 28,376.06)	0.788
**FRAP (mmol Fe**^**+2**^**/d)**	36.95 (16.49)	38.47 (18.69)	43.36 (16.95)	0.733	47.87 (20.43)	45.07 (18.84)	45.95 (10.61)	0.813
**TRAP (mmol TE/d)**	24.89 (15.86, 30.73)	23.32 (15.85, 38.23)	27.03 (15.54, 43.58)	0.816	31.89 (13.29)	31.42 (15.18)	29.27 (7.86)	0.868

Data are presented as mean (SD) or median (25 and 75 percentiles). ^a^Analysis of variance for continuous variables and χ2 test for categorical variables. *WC* Waist circumference, *SES* Socio-economic status, *FM* Fat mass, *FFM* Fat free mass, *FADS* Fatty acid desaturase, *ORAC* Oxygen Radical Absorbance Capacity, *FRAP* Ferric Reducing Ability of Plasma, *TRAP* Total Radical-Trapping Antioxidant Parameter

### Associations of *FADS*2 rs174583 with dietary NEAC

Table 2 presents sex-stratified analysis for the relationship between dietary NEAC and *FADS* rs174583 genotypes. No statistically significant association was found between NEAC indicators and *FADS*2 polymorphism; neither in crude nor in the adjusted models among both female and male subjects.

**Table 2:** Odd’s ratio (OR) and confidence interval (CI) for the association between NEAC tertiles and *FADS*2 rs174583 genotypes

	**Men**			**Women**		
	**CC**	**CT**	**TT**	**CC**	**CT**	**TT**
**ORAC. Total score**						
**Crude**	1(Ref.)	1.00 (1.00–1.00)	1.00 (1.00–1.00)	1(Ref.)	1.00 (1.00–1.00)	1.00 (1.00–1.00)
**Model 1**^a^	1(Ref.)	1.00 (1.00–1.00)	1.00 (1.00–1.00)	1(Ref.)	1.00 (1.00–1.00)	1.00 (1.00–1.00)
**Model 2**^b^	1(Ref.)	1.00 (1.00–1.00)	1.00 (1.00–1.00)	1(Ref.)	1.00 (1.00–1.00)	1.00 (1.00–1.00)
**FRAP. Total score**						
**Crude**	1(Ref.)	0.99 (0.97–1.02)	0.98 (0.96–1.03)	1(Ref.)	1.01 (0.98–1.04)	1.02 (0.97–1.07)
**Model 1**^a^	1(Ref.)	0.99 (0.97–1.01)	0.99 (0.96–1.04)	1(Ref.)	1.01 (0.98–1.04)	1.02 (0.97–1.08)
**Model 2**^b^	1(Ref.)	0.99 (0.97–1.02)	1.00 (0.96–1.03)	1(Ref.)	1.01 (0.98–1.04)	1.02 (0.97–1.08)
**TRAP. Total score**						
**Crude**	1(Ref.)	1.00 (0.97- 1.03)	0.99 (0.93–1.04)	1(Ref.)	1.00 (0.97–1.04)	1.02 (0.96–1.08)
**Model 1**^a^	1(Ref.)	1.00 (0.97–1.04)	0.98 (0.93–1.04)	1(Ref.)	1.01 (0.97–1.04)	1.03 (0.96–1.11)
**Model 2**^b^	1(Ref.)	1.00 (0.97–1.04)	0.98 (0.93–1.04)	1(Ref.)	1.01 (0.97–1.05)	1.03 (0.96–1.11)

The multivariate multinomial logistic regression was used for estimation of ORs and confidence interval (CI). ^a^Adjusted for age, physical activity and socio-economic status. ^b^Additionally adjusted for waist circumference

### Differences in distribution of MetS and means of cardio-metabolic variables between *FADS*2 rs174583 genotypes according to gender

Sex-stratified analysis for the association between laboratory and clinical parameters and *FADS* rs174583 genotypes are displayed in Table 3. As shown in this Table, male TT-genotype carriers had higher mean values of TG (*P* = 0.037) and AIP (*P* = 0.041) compared to other genotype categories (CT, CC), whereas no significant associations were revealed among female participants.

**Table 3:** Clinical and biochemical characteristics of study participants according to rs174583 genotypes

	**Men**				**Women**			
	**CC**	**CT**	**TT**	***P***^a^	**CC**	**CT**	**TT**	***P***^a^
**LDL-C, (mg/dl)**	116.78 (24.67)	120.63 (30.85)	114.36 (28.81)	0.755	113.64 (30.80)	118.61 (35.12)	133.27 (28.73)	0.435
**HDL-C, (mg/dl)**	42.14 (5.35)	43.62 (7.60)	40.10 (7.65)	0.303	48.50 (11.03)	48.90 (7.78)	42.00 (13.74)	0.252
**Cholesterol, (mg/dl)**	184.20 (26.95)	189.67 (36.44)	189.90 (32.69)	0.744	182.79 (34.08)	188.48 (35.55)	198.50 (34.51)	0.591
**TG, (mg/dl)**	111.00 (81.00, 153.00)	111.00 (88.00, 140.00)	171.00 (132.50, 247.00)	**0.037**	80.00 (68.75, 128.00)	99.50 (73.75, 130.25)	107.00 (86.75, 152.00)	0.551
**AIP**	0.07 (0.23)	0.06 (0.23)	0.26 (0.21)	**0.041**	-0.07 (0.28)	-0.06 (0.20)	0.08 (0.28)	0.369
**Glucose, (mg/dl)**	91.00 (85.00, 97.00)	92.00 (87.00, 97.00)	96.50 (87.00, 109.25)	0.633	90.00 (86.00, 94.00)	89.00 (85.00, 97.25)	93.00 (82.00, 109.50)	0.836
**Insulin, U/mL**	11.40 (9.00, 19.30)	13.20 (8.60, 24.80)	10.45 (5.58, 13.98)	0.144	17.45 (9.28, 27.25)	13.15 (8.55, 19.15)	20.10 (10.75, 42.55)	0.265
**HOMA-IR**	2.68 (2.00, 4.79)	3.22 (1.95, 5.30)	2.24 (1.09, 3.38)	0.246	3.89 (1.87, 6.25)	3.02 (1.94, 4.79)	4.52 (2.45, 10.93)	0.326
**QUICKI**	0.33 (0.03)	0.32 (0.03)	0.34 (0.03)	0.285	0.33 (0.04)	0.32 (0.03)	0.31 (0.04)	0.426
**SBP (mmHg)**	114.63 (23.04)	117.82 (12.45)	120.00 (14.14)	0.618	112.29 (13.87)	113.52 (13.32)	111.83 (7.76)	0.913
**DBP (mmHg)**	75.54 (15.88)	76.41 (11.12)	76.50 (9.14)	0.955	76.25 (10.40)	77.14 (12.44)	71.33 (7.66)	0.514
**Mets (%)**	41.2	38.2	20.6	0.303	43.8	50.0	6.2	0.478

Data are presented as mean (SD) or median (25 and 75 percentiles). ^a^Analysis of variance for continuous variables and χ2 test for categorical variables. *WC* Waist circumference, *HOMA-IR* Homeostasis model assessment of insulin resistance, *LDL-C* Low density lipoprotein cholesterol, *HDL* High-density lipoprotein-cholesterol, *SBP* Systolic blood pressure, *DBP* Diastolic blood pressure, *TG* Triglyceride, *QUICKI* Quantitative insulin sensitivity check index, *AIP* Athrogenic index of plasma, *MetS* Metabolic syndrome

### Sex-stratified interaction analysis between dietary NEAC and *FADS*2 rs174583 in relation to cardio-metabolic risk factors

**Fig. 1:**
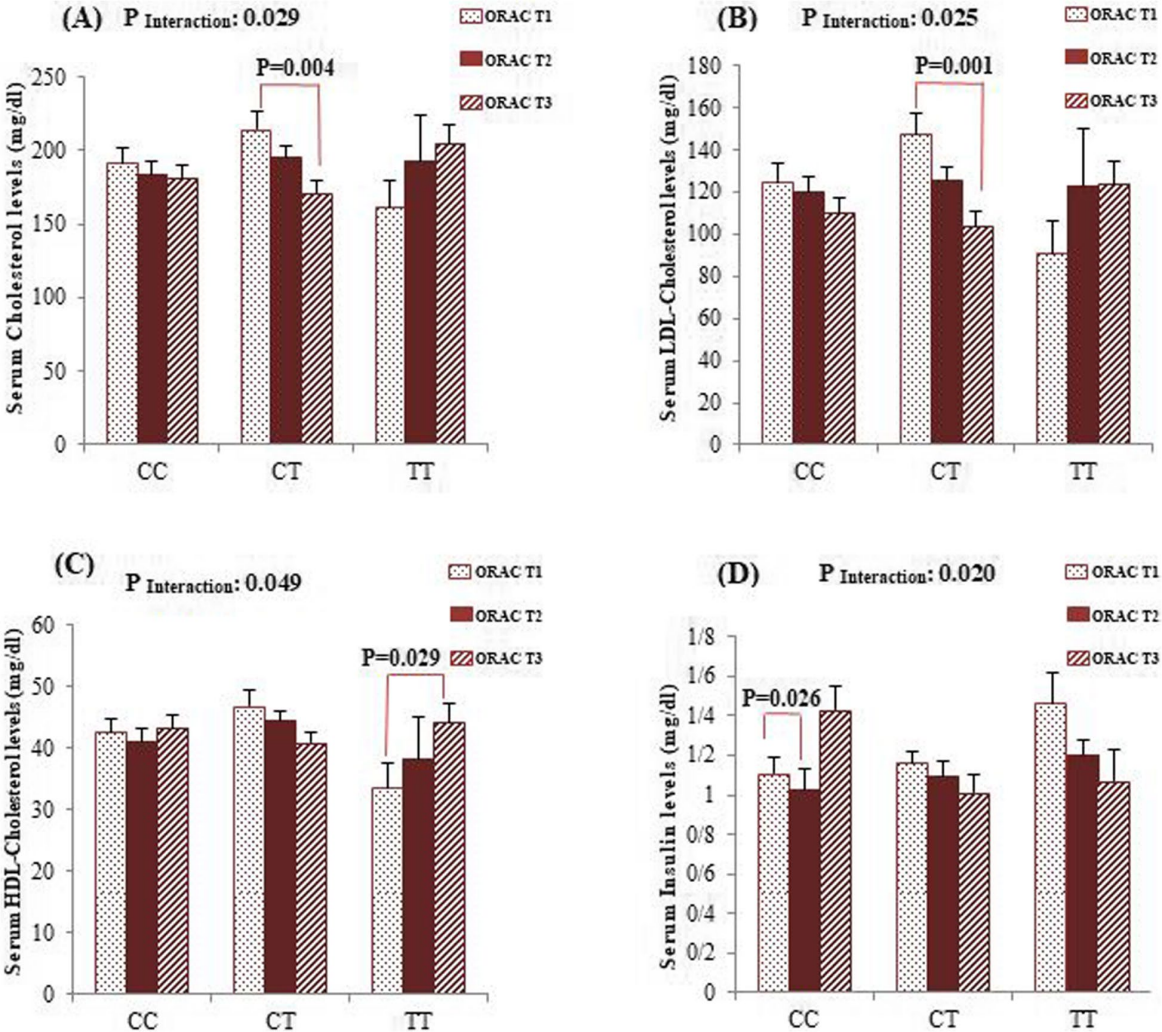
Interaction between FADS2 rs174583 and dietary ORAC on serum concentration of cholesterol (**A**), LDL-C (**B**) and HDL-C (**C**) among men. Interaction between FADS2 rs174583 and dietary ORAC on serum insulin level (**D**)

**Fig. 2:**
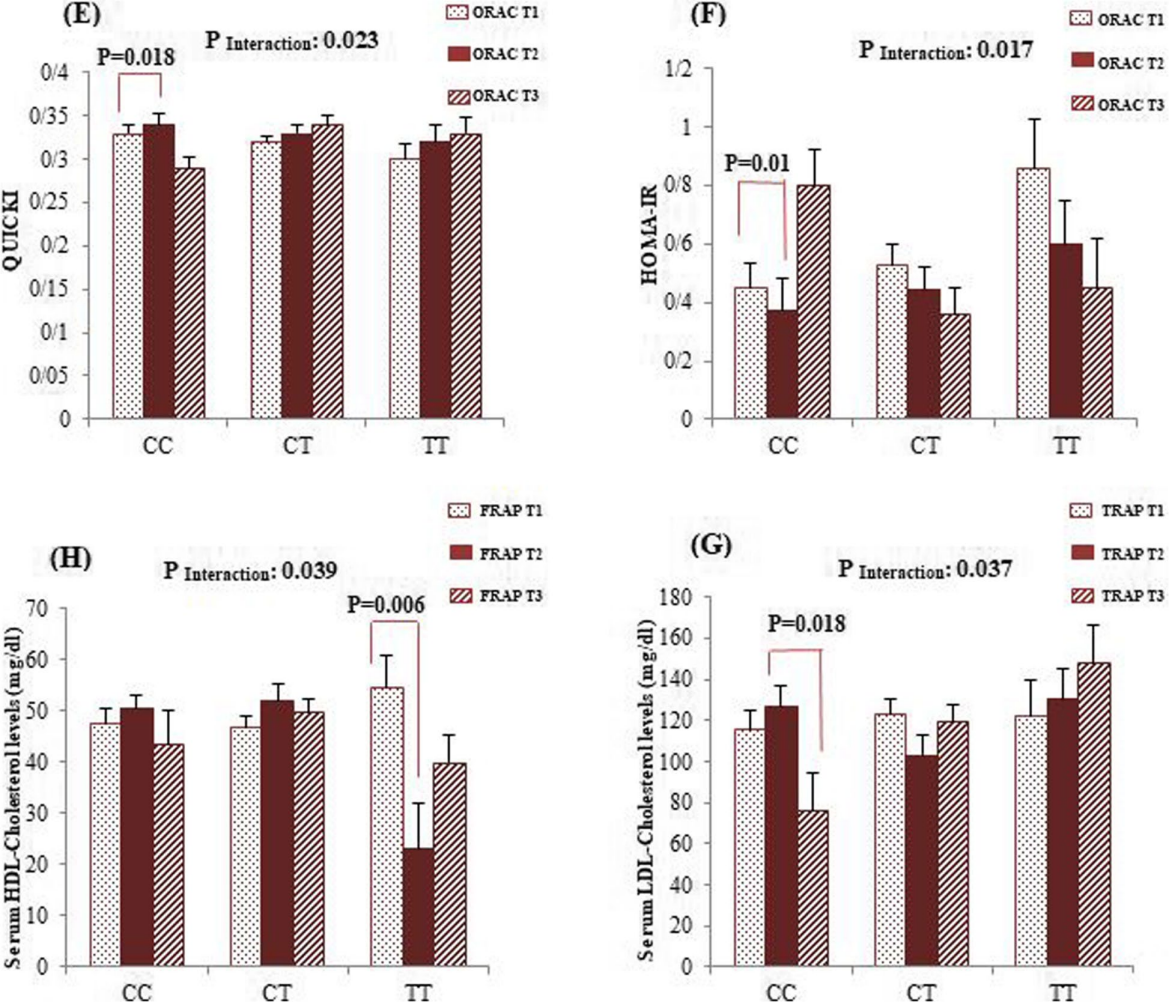
Interaction between FADS2 rs174583 and dietary ORAC on QUICKI (**E**) and HOMA-IR among women (**F**). Interaction between FADS2 rs174583 and dietary TRAP on serum LDL-C level among women (**G**) and all *P*-values of interactions were adjusted for age, WC, physical activity and socio-economic status. Interaction between FADS2 rs174583 and dietary FRAP on serum concentration of HDL-C among women in crude model (**H**). The bars indicate mean. Error bars: SE of means

We performed sex-stratified covariance analyses to verify whether adherence to the dietary NEAC modifies the association of the *FADS*2 rs174583 polymorphism with cardio-metabolic risk factors. Subsequently, statistically significant interactions are illustrated in Figs. 1 and 2. Among male participants, after adjustment for confounding variables (age, physical activity, SES and WC), the interactions between rs174583 polymorphism and adherence to dietary ORAC on serum cholesterol (*P*_Interaction_ = 0.029), LDL-C (*P*_Interaction_ = 0.025) and HDL-C levels (*P*_Interaction_ = 0.049) were statistically significant. In male CT-genotype carriers, the serum levels of cholesterol (*P* = 0.004) and LDL-C (*P* = 0.001) in subjects who assigned to the highest tertile of ORAC were significantly lower than subjects in the first category. Additionally, male subjects with homozygous minor allele genotype had higher means of HDL-C when had the highest compliance with dietary ORAC (*P* = 0.029). From our analyses among women, the dietary ORAC-rs174583 interactions were statistically significant for serum insulin concentrations (*P*_Interaction_ = 0.020), QUICKI (*P*_Interaction_ = 0.023) and HOMA-IR (*P*_Interaction_ = 0.017) even after adjustment for confounding variables. According to these interactions, in CC genotype group, serum insulin levels (*P* = 0.026) and HOMA-IR (*P* = 0.010) were significantly lower in female participants who were assigned in the second tertile of dietary ORAC in comparison with the first category. In contrast, the highest QUICKI was observed in the female CC-genotype carriers who had moderate compliance with the dietary ORAC (*P* = 0.018). Moreover, we found a relevant interaction between adherence to the dietary FRAP in relation to serum HDL-C concentrations (*P*_Interaction_ = 0.039) among women, whereas adjustment for potential confounders eliminated this significant interaction. In addition, the dietary TRAP modified the association between *FADS*2 variant and change in LDL-C levels (*P*_Interaction_ = 0.037); the homozygous wild-type (CC) women who placed in the top tertile of TRAP had significantly the lowest LDL-C levels than those in the second tertile (*P* = 0.018).

## Discussion

To the best of our knowledge, this is the first study that has investigated the interactions between *FADS*2 gene polymorphism (rs174583) and dietary NEAC in relation to cardio-metabolic risk factors. We documented the interactions of *FADS*2 gene rs174583 SNP with dietary NEAC (ORAC), in changing serum lipid profiles among male subjects; minor allele carriers who had the highest adherence to the NEAC showed a better metabolic profile (lower TG and LDL-C and higher HDL-C). Among female subjects, dietary ORAC intake modified the relationship of *FADS*2 variant with glycemic indices; the highest QUICKI and the lowest HOMA-IR and serum insulin levels were observed in the CC homozygote carriers with moderate compliance with the dietary ORAC. Additionally, being in the highest tertiels of TRAP could show beneficial effects in decreasing LDL-C in homozygous females for the major allele (CC).

The frequency of T minor allele of *FADS*2 rs174583 polymorphism was 36% which was lower than what had been previously reported in European (HapMap database) and Taiwanese population [49]. Differences in study design, sample size, dietary habits, lifestyle and population characteristics such as ethnicity may partly explain the discrepancies in allele frequencies among different studies.

Although no association was found between psychiatric variables and rs174583 in the current research, it has been shown that minor allele of the *FADS*2 rs174583 has a positive relationship with perinatal depressive disorders risk among Canadian women [50]. Besides, it is generally supposed that abnormalities exist in the composition of fatty acids particularly ω-3 PUFAs in human tissues, play an important role in the pathogenesis of both mood disorders and chronic diseases such as CVDs [51, 52]. In the present study, we documented significant differences in serum TG level and AIP between different genotypes; TT genotype group had a higher TG and AIP compared to other genotype categories, which were in accordance with some previous studies [53, 54]. For instance, a recent publication by Mazoochian et al. reported a higher level of TG in minor allele homozygote group (TT) of rs174583 than CT heterozygote carriers with T2D [53]. Despite the fact that exact mechanisms contribute to the association between *FADS* genetic variants and disease development remain unknown; current evidence has suggested that *FADS* genetic variations may impair desaturases leading to changes in n-3/n-6 PUFA status which has been associated with the risk of several chronic diseases [49, 55].

Generally, the present study showed that improving adherence to dietary NEAC (reflected in ORAC and TRAP) can significantly attenuate the genetic association with cardio-metabolic risk factors. These findings propose that male carriers of the minor allele (CT and TT) and female homozygous carriers of the major allele (CC) of *FADS*2 rs174583 appear to be protected against increase in metabolic risk factors when they consume antioxidant-rich foods. As mentioned above, some of the significant interactions were found to be sex-specific. There is no clear mechanism to explain these gender-dependent heterogeneities; however, difference in regional depots of adipose tissue and hormonal status may be reasons for these sex-based differences [56]. Besides, it seems that the non-significant higher dietary NEAC score may help to describe, in part, these inconsistences. For example, men with TT genotype had a non-significant higher median of ORAC compared with other genotypes (CT and CC). A wealth of evidence has accumulated about gender differences in dietary intakes and eating behavior; compared to men, women had healthier dietary pattern, lifestyle and food choices [57]. To date, there is no study in the literature regarding the interactions between *FADS*2 rs174583 and dietary antioxidant intakes, as measured by overall NEAC scores, in relation to health outcomes with which we can directly compare our findings. Nonetheless, our results are supported by some observational studies in which a modulation by diet on the association of the *FADS*1 and *FADS*2 genes with metabolic disorders like MetS has been reported [55, 58]. In a recent cross-sectional genome wide association study (GWAS) on Korean population, Park et al. revealed statistically significant interactions between total fat intake and the *FADS*1 rs174547 and haplotype of *FADS*1 rs174547 and *FADS*2 rs2845573 on MetS risk and it seemed that intermediate fat intake protected carriers of the *FADS*1 major alleles against the risk of MetS [58]. Additionally, these findings are consistent with our previous study in which good adherence to the dietary NEAC could attenuate the association of melanocortin 4 receptor (MC4R) gene polymorphism with some of metabolic risk factors [59]. Since the majority of studies which have documented a protective role for dietary NEAC against obesity [10] and other health outcomes such as MetS [60], cancers [13] and T2D [12], it is not surprising that high intakes of NEAC neutralize detrimental effects of greater genetic predisposition to cardio-metabolic risk factors in *FADS*2 minor allele carriers that this means these individuals are more likely to respond to high intakes of dietary antioxidants. These protective influences of antioxidant-rich foods on metabolic status may happen through increasing insulin sensitivity and thermogenesis, regulation of appetite and modification of lipids and carbohydrate metabolism [61]. Nonetheless, in the present study, no significant difference in mean of appetite score was found between rs174583 genotypes and different tertiles of NEAC (data have not shown). It is worth noting that regulation of appetite is a complex mechanism involving the connections between hypothalamus and the brainstem within the central nervous system (CNS), gastrointestinal tract and adipose tissue [62]. Thus, it didn’t appear that antioxidant-rich foods lonely have favorable effects on appetite in our study. However, it has been shown that high intake of foods rich in bioactive redox substances such as vegetables and fruits exert health benefits not only by protecting against oxidative damages, but also through providing magnesium, fiber, potassium, and other phytochemicals which may have synergetic effects on prevention of human diseases [63].

The present study had some potential limitations that should to be taken into account when interpreting the results. First, since it was a cross-sectional, causation cannot be inferred, while these results can provide the hypothesis that can then be assessed more rigorously via prospective cohort or other studies. Second, the relatively small sample size of studied subjects was a serious and major limitation of the present research which may restrict the achievement of an adequate statistical power. Thus, our results need to be taken with caution and replicated in large longitudinal studies. Third, as obese adults tend to underreport their dietary intakes, it may cause misclassifications in dietary variables and this potential bias may consequently result in an underestimate of the true effect. For this reason, subjects with extreme dietary intake values were removed from the analyses. Forth, although we controlled carefully for several confounders in the analyses, residual unknown confounders that might distort the findings could not be fully eliminated. Fifth, to calculate NEAC, the international databases were used as there were no NEAC values for the local foods. It should be notice that due to different growing conditions and geographic location, using antioxidant values from other countries may not be appropriate and these figures may not be the same for Iranian foods. Nevertheless, it has been shown that the assessment of dietary NEAC through FFQ is a valuable and valid measure in epidemiological studies [63, 64]. Sixth, the assessment of dietary NEAC does not take into account metabolism or antioxidant bioavailability. Furthermore, other variants within the *FADS* gene cluster, as well as variants in other genes contribute to the pathogenesis of obesity and related- metabolic phenotypes. Last, since this study was carried out among population from Tabriz city, the generalizability cannot be taken for granted.

## Conclusion

Our findings for the first time suggest that the dietary NEAC intakes may modify the association of the genetic variation in *FADS*2 with cardio-metabolic risk factors. So, our results provide more evidence that recommendation of antioxidant-rich foods can be a suitable strategy for disease prevention and health promotion particularly in people with susceptible genotypes; however replication in large cohort of other population is required.

## Supplementary Information

**Additional file 1.**

## Data Availability

Data used in the study cannot be deposited publically because of some institutional restrictions; however, data could be available by reasonable from the corresponding author.

## Abbreviations

AIP: The atherogenic index of plasma
ANOVA: Analysis of variance
BIA: Bioelectrical impedance analysis
BMI: Body mass index
CVD: Cardiovascular disease
DASS-21: Depression, Anxiety and Stress Scale-21 Items
DBP: Diastolic blood pressure
D5D: Delata-5-desaturase
D6D: Delta-6-desaturase
ELISA: Enzyme-linked immunosorbent assay
*FADS*: Fatty acid desaturases
FCT: Food Composition Table
FFQ: Food-frequency questionnaire
FRAP: Ferric reducing ability of plasma
GWAS: Genome wide association study
HDL-C: High-density lipoprotein cholesterol
HOMA-IR: Homeostasis model assessment-insulin resistance index
IPAQ: International Physical Activity Questionnaire
LDL-C: Low-density lipoprotein-cholesterol
MC4R: Melanocortin 4 receptor
MetS: Metabolic syndrome
NEAC: Non-enzymatic total antioxidant capacity
ORAC: Oxygen radical absorbance capacity
PUFA: Polyunsaturated fatty acids
PCR–RFLP: Polymerase chain reaction-restricted length polymorphism
QUICKI: Quantitative insulin sensitivity check index
ROS: Reactive oxygen species
SBP: Systolic blood pressure
SES: Socioeconomic status
SNPs: Single‐nucleotide polymorphisms
SPSS: Statistical Package for Social Sciences
TAC: Total antioxidant capacity
TG: Triglyceride
TRAP: Total radical-trapping antioxidant parameters
T2D: Type 2 diabetes
VAS: Visual analog scale
