# Supplementary material for: Interplay between fatty acid desaturase2 (FADS2) rs174583 genetic variant and dietary antioxidant capacity: cardio-metabolic risk factors in obese individuals
Source: BMC Endocr Disord. 2022 Jun 30;22:167. doi: 10.1186/s12902-022-01075-7 (PMC9245241; doi:10.1186/s12902-022-01075-7)
Supplement: Supplementary file 1 — Additional file 1. [file 12902_2022_1075_MOESM1_ESM.docx]

**Supplementary Material**

**Interplay between fatty acid desaturase2 (*FADS*2) rs174583 genetic variant and dietary antioxidant capacity: cardio-metabolic risk factors in obese individuals**

**Biochemical measurements**

Venous blood samples were collected in the morning after overnight fast and then centrifuged at 4500 rpm, for 10 min at 4°C to obtain the serum samples. Aliquots were stored at -80∘C until they were analyzed. Total cholesterol, triglyceride (TG), high-density lipoprotein cholesterol (HDL-C) and glucose concentrations were measured by enzymatic colorimetric method using commercially available kits (Pars Azmoon, Tehran, Iran). Serum low-density lipoprotein-cholesterol (LDL-C) was calculated as described by Friedewald et al equation (1). Serum concentrations of insulin were analyzed through commercially available enzyme-linked immunosorbent assay (ELISA) kits (Bioassay Technology Laboratory, Shanghai Korean Biotech, Shanghai City, China). The homeostasis model assessment-insulin resistance index (HOMA-IR) and quantitative insulin sensitivity check index (QUICKI) were obtained according to the methods of Matthews et al. and Katz et al., respectively (2, 3). The atherogenic index of plasma (AIP) was estimated as logarithmic transformation of TG to HDL-C ratio (4).

**Genotyping**

The extraction of genomic DNA from blood samples was carried out with the use of standard phenol/chloroform method. The quality and quantity of the extracted DNA were assessed using Nano Drop ND-1000 spectrophotometer. In the present study, subjects were genotyped for the SNP rs174583, which is located in the position chr11:61842278 in the intron region of *FADS*2 gene, using polymerase chain reaction-restricted length polymorphism (PCR–RFLP) technique. Template primers used for the PCR amplification of the rs174583 were as follows: forward: 5′ AGGAAGCAGACCACAGAGTC 3′; reverse, 5′ TCCTTCGTCTGGTGTCTCAG 3′. The PCR reaction was optimized in a 10 μl total volume containing 2 μl extracted DNA, 5 μl master mix (Ampliqon; Denmark), 1 μl primers and 2 μl distilled water. The PCR amplification was carried out in a DNA thermocycler (BIO RAD T100 Thermal Cycler) with an initial denaturation at 95 °C for 10 min, followed by 35 cycles of denaturation at 95 °C for 15 s, annealing at 60 °C for 20 s, extension at 72 °C for 50 s, and the final extension at 74 °C for 10 min. Based on the restriction sites on the sequence of the amplified DNA, TauI (cat. Num ER1652, USA) was used as restriction enzyme for digestion. Then, the digested PCR products were subjected to electrophoresis on 1.5 % agarose gel, stained with green viewer and visualized on a Gel Doc system (U.V.P Company, Cambridge, UK). To determine the length of the digested products, a 50 bp ladder was applied. Finally, fragments containing three possible genotypes were distinguished: uncut homozygous TT (572 bp), cut heterozygous CT (192, 380 and 572 bp) and cut homozygous CC (192, 380) (Sup. Figure 1, 2).


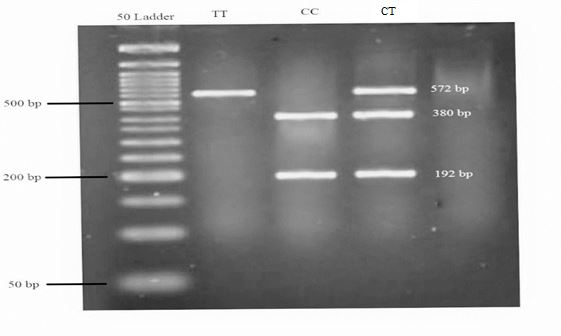


**Sup. Figure 1.** Genotyping of FADS2 rs174583 variant by TauI PCR-RFLP analysis. A 50 bp Ladder was applied to determine the length of the digested products. CC= homozygous wild-type (380 and 192 bp), CT= heterozygous mutated (572, 380, and 192 bp).TT= homozygous mutated (572 bp).

**
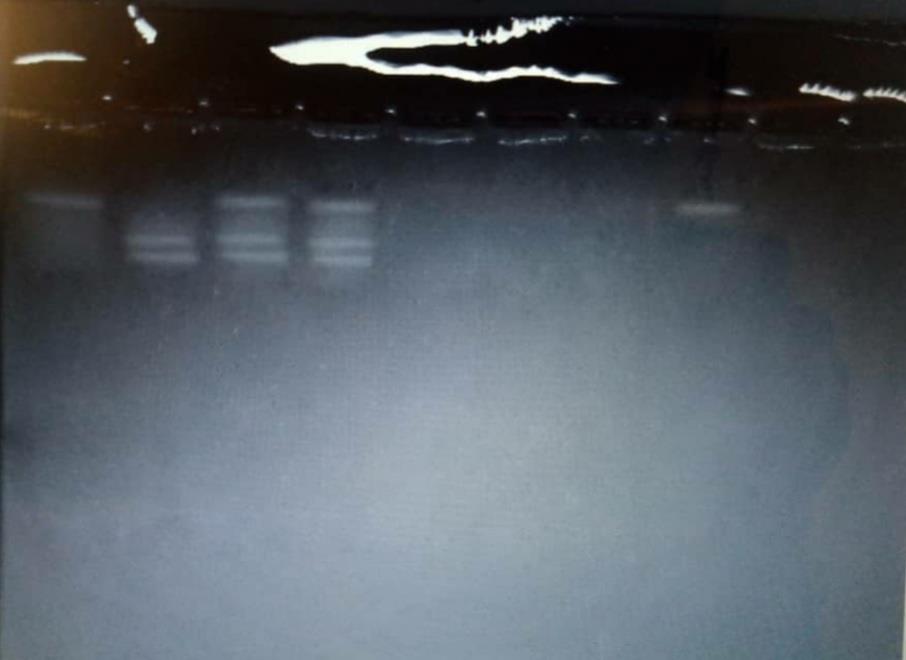
**  **
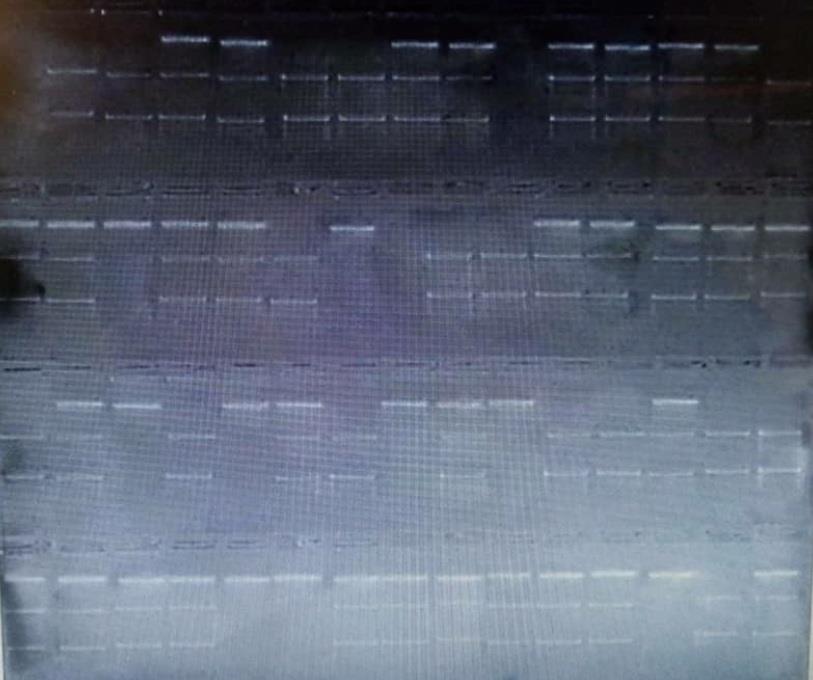
**

**
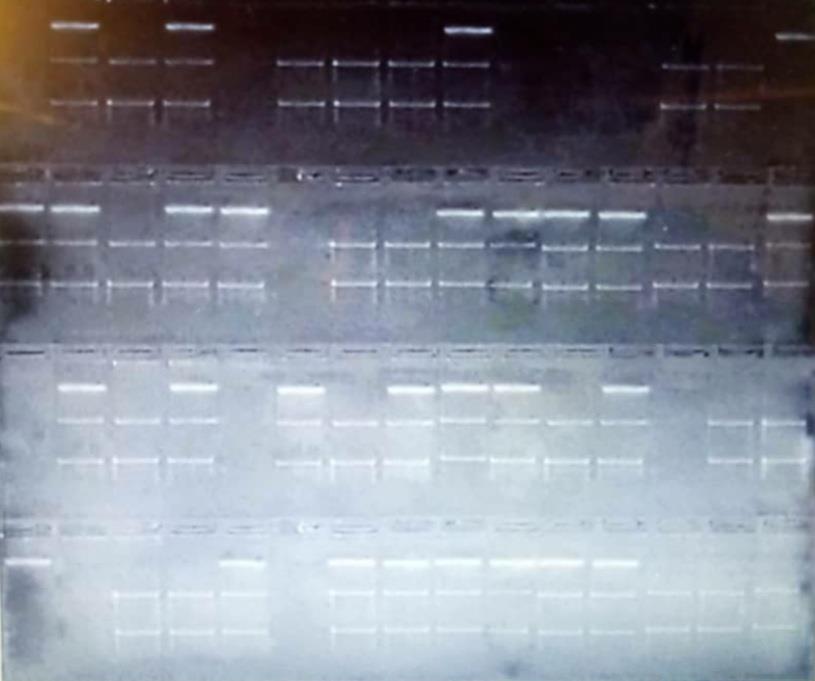

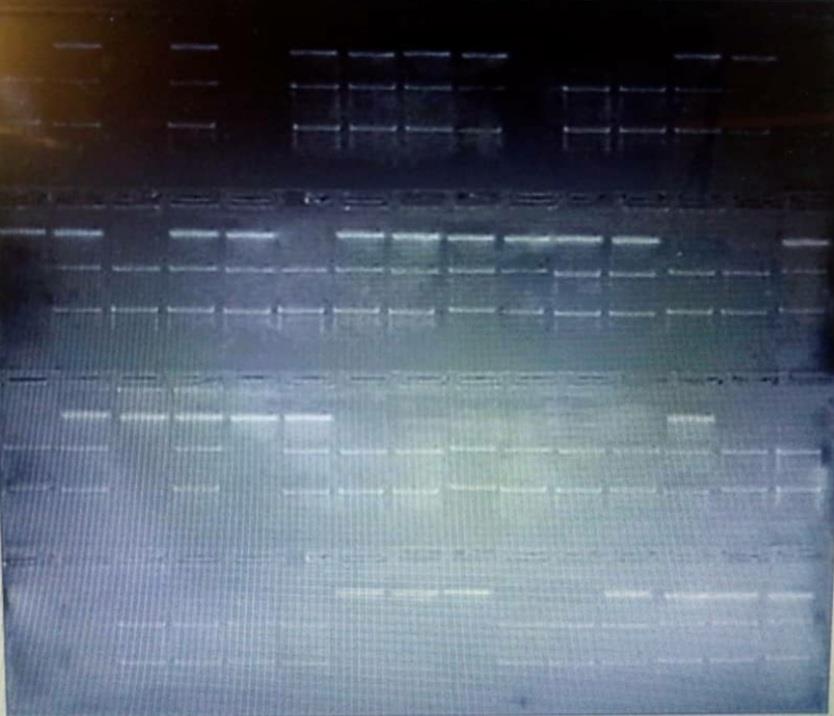
**

**Supp. Figure 2.** Original, unprocessed **full length gels and blots of** FADS2 rs174583 variant by TauI PCR-RFLP analysis

**References**

1. Matthews DR, Hosker JP, Rudenski AS, Naylor BA, Treacher DF, Turner RC. Homeostasis model assessment: insulin resistance and beta-cell function from fasting plasma glucose and insulin concentrations in man. Diabetologia. 1985;28(7):412-9, doi: 10.1007/bf00280883.
2. Katz A, Nambi SS, Mather K, Baron AD, Follmann DA, Sullivan G, et al. Quantitative insulin sensitivity check index: a simple, accurate method for assessing insulin sensitivity in humans. The Journal of clinical endocrinology and metabolism. 2000;85(7):2402-10, doi: 10.1210/jcem.85.7.6661.
3. Wu TT, Gao Y, Zheng YY, Ma YT, Xie X. Atherogenic index of plasma (AIP): a novel predictive indicator for the coronary artery disease in postmenopausal women. Lipids in health and disease. 2018;17(1):197, doi: 10.1186/s12944-018-0828-z.
